# Development of Efficient and Stable Inverted Bulk Heterojunction (BHJ) Solar Cells Using Different Metal Oxide Interfaces

**DOI:** 10.3390/ma6125796

**Published:** 2013-12-10

**Authors:** Ivan Litzov, Christoph J. Brabec

**Affiliations:** 1 Institute of Materials for Electronics and Energy Technology(I-MEET), Friedrich-Alexander University of Erlangen-Nuremberg, Martensstrasse 7, Erlangen 91058, Germany; E-Mail: christoph.brabec@ww.uni-erlangen.de; 2 Bavarian Center for Applied Energy Research (ZAE Bayern), Haberstrasse 2a, Erlangen 91058, Germany

**Keywords:** sol-gel methods, metal oxide interfaces, inverted structure, device stability

## Abstract

Solution-processed inverted bulk heterojunction (BHJ) solar cells have gained much more attention during the last decade, because of their significantly better environmental stability compared to the normal architecture BHJ solar cells. Transparent metal oxides (MeO*_x_*) play an important role as the dominant class for solution-processed interface materials in this development, due to their excellent optical transparency, their relatively high electrical conductivity and their tunable work function. This article reviews the advantages and disadvantages of the most common synthesis methods used for the wet chemical preparation of the most relevant *n*-type- and *p*-type-like MeO*_x_* interface materials consisting of binary compounds A*_x_*B*_y_*. Their performance for applications as electron transport/extraction layers (ETL/EEL) and as hole transport/extraction layers (HTL/HEL) in inverted BHJ solar cells will be reviewed and discussed.

## 1. Introduction

Solar cells are considered an ideal technology for renewable power generation, as they directly convert solar energy into electricity without any “green-house” gas emission. Today, still, solar cells based on Si dominate the photovoltaic (PV) market [1]. With the discovery of high conductivity in perylene-iodine complexes in 1954, organic semiconductors have become intensively researched. The unique properties of organic semiconductors, like flexibility, thinness and simple fabrication processes, enable the potential applications for organic solar cells (OSCs) and organic light emitting diodes (OLEDs) [2]. However one of the major breakthroughs in the field of OSC was the discovery of bulk heterojunction (BHJ) composites, resulting in two different prototype architectures: on the one hand, the “normal structure” architecture, traditionally requiring reactive cathode materials, like Ca or LiF [3,4,5,6,7,8,9,10,11,12,13,14], and, on the other hand, the “inverted structure” [15,16,17,18,19,20,21,22,23,24,25,26,27,28,29], allowing one to work with air stable cathode materials, like indium tin oxide (ITO) or Ag. Therefore, inverted architecture BHJ solar cells provide a good strategy for improved air stability. The interface materials play an essential role in designing efficient and stable BHJ solar cells. MeO*_x_* have been reported not only as an electron/hole transport layer (ETL/HTL) or an electron/hole extraction layer (EEL/HEL), but also as an electrical contact to a less air-sensitive high work function metal, e.g., Ag or Al (Au) [22,23,24,25].

In general, MeO*_x_* have good optical transparency and sufficient electrical conductivity. Today, the focus of scientists is on the processing of highly transparent and conductive thin films based on n-type- and p-type-like semiconductor metal oxides [23,30,31,32]. Most of these MeO*_x_* are binary compounds, like ZnO, TiO_2_, SnO_2_, In_2_O_3_, MoO_3_, WO_3_ or V_2_O_5_, consisting of one metallic element. One advantage of using binary compounds as interface materials is that their chemical composition in film depositions is relatively easy to control, compared to the ternary compounds and multicomponent oxides [33].

Metal oxides can be prepared in various sizes and geometries, but one of the greatest challenges remains the precise control of the particle size, shape, crystalline structure and processing properties.

A great number of synthesis methods were tested over the last several years [34,35,36,37,38,39,40,41,42,43,44,45,46,47,48,49,50,51,52,53,54,55,56,57,58,59,60,61,62,63,64], which all allow access to MeO*_x_* nanomaterials with uniform crystal structure, form and varied composition. The liquid phase synthesis methods are more versatile with regards to controlling the structural and morphological properties of the products, compared to, for example, the gas phase processes [39]. Potential pathways in liquid phase syntheses include a precipitation reaction, an aqueous or non-hydrolytic sol-gel process [38,41,56,57,61] and hydrothermal [49,59] or solvothermal methods [53,58]. The synthesis of the desired metal oxide nanoparticles requires, in some cases, a combination of these techniques. Aqueous sol-gel methods were used for decades for the production of highly monodisperse colloids, despite their limitations concerning the low crystallinity. Additionally, the aqueous synthesis pathways are very demanding, due to the higher reactivity of metals in the starting precursors and the double functionality of water as the solvent and ligand. The slightest variations of reaction conditions may leads to different particle morphologies and, therefore, complicate the use of these synthesis routes for industry applications [57].

In this review, we will summarize the most frequently published chemical methods for the synthesis of transition metal oxides. A focus is placed on the classical sol-gel methods (precipitation reactions) [34,35,37,38,40,41,43,44,45,52,64] and the so-called non-hydrolytic sol-gel methods [49,53,56,57,61] at moderate temperatures without surfactants. In the second part of this review, we will discuss the application of the corresponding MeO*_x_* as interface layers in the inverted BHJ organic solar cells.

## 2. Comparison between the Classical Sol-Gel Method (Precipitation Reactions) and the Non-hydrolytic Sol-Gel Method

In this section, we will discuss the principles of the sol-gel process, with particular emphasis on its potential for transition metal oxide (MeO*_x_*) synthesis via the classical sol-gel method (precipitation reactions) and the non-hydrolytic sol-gel method.

**Figure 1:**
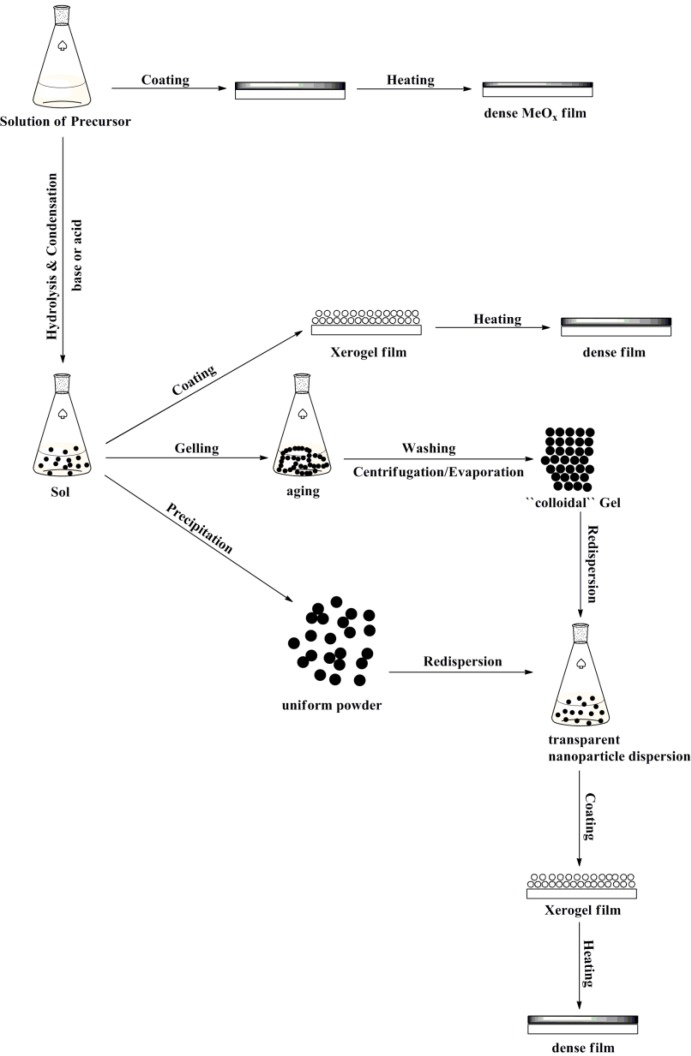
Various sol-gel options to control the final morphology of the different product.

In general, the sol-gel process consists of the following steps: (1) preparation of a homogeneous solution, either by dissolution of the metal organic precursors in an organic solvent or by dissolution of inorganic salts in an organic solvent or water; (2) conversion of the homogeneous solution into a sol by treatment with a suitable reagent (generally base); (3) aging; (4) shaping; and (5) thermal treatment or sintering [65] (Figure 1).

### 2.1. Classical Sol-Gel Method

The classical sol-gel method (precipitation reactions) is the conversion of a precursor solution into an inorganic solid via inorganic polymerization reactions induced by strong bases (KOH; NaOH; LiOH∙H_2_O; LiOH). The starting compound is either an inorganic metal salt (chloride; nitrate; sulfate, and so on) or a metal organic compound, such as acetate [Equation (1)] [65]. However, this method has several disadvantages in which the formation of condensed species from alcoholic solutions of inorganic salts or metal organic compound occurs by adjusting the pH, by increasing the temperature or by changing the oxidation state. The chemistry of transition metal ions is complicated, because of the formation of a large number of oligomeric species. The nature of these species depends on the counter anions, which are able to coordinate the metal ion and initiate new molecular precursors with different chemical reactivity towards hydrolysis and condensation. These counter ions can influence the morphology, the structure and even the chemical composition of the resulting solid phase. Furthermore, the removal of these anions from the final metal oxide product is frequently a big challenge [65].

MX_2_ + 2BH → MO_(S)_ + 2B^+^ + 2X^−^ + H_2_O (1)

where X = (CH3COO^−^; Cl^−^; NO_3_^−^); B = (Na^+^; K^+^; Li^+^); solvent: methanol; ethanol; *iso*-propanol. This equation is the inorganic polymerization reactions induced by a base.

These challenges can be addressed by using metal organic compounds (e.g., metal alkoxides), which can be dissolved in organic solvents (sMeO*_x_*). These precursor solutions provide highly homogeneous mixtures and can be directly deposited and thermally converted into the corresponding oxide (See Figure 1).

### 2.2. Non-Hydrolytic Sol-Gel Method

The main problem of the classical sol-gel method based on hydrolysis and condensation of molecular precursors is the control over the reaction rate. For most transition metal oxide precursors, these reactions are very fast, resulting in the loss of morphological and also structural control over the final material composition. One possibility to decrease and to adjust the reactivity of the precursors is the use of organic additives, like amines, β-diketones, carboxylic acids or functional alcohols, which act as chelating ligands and modify the reactivity of the precursors [65]. Nevertheless, general predictions for the various reaction paths between the central metal ions with different ligands are still under intense research.

In the non-hydrolytic sol-gel process, divided into surfactant-directed and solvent-controlled approaches, the transformation of the precursor takes place in an organic solvent under exclusion of water or a base. Interestingly, independent of whether surfactants are used or not, only five distinct condensation reaction mechanisms partly explain the formation of the metal–oxygen–metal (M–O–M) bond. These condensation steps are: (1) alkyl halide elimination; (2) ether elimination; (3) ester and amide elimination; (4) C–C coupling of benzylic alcohols and alkoxide molecules; and (5) aldol/ketimine condensation [57,61].

According to Vioux [36], in non-hydrolytic procedures using hydrated metal oxide precursors and/or water (H_2_O) produced *in situ*, a hydrolytic reaction pathway cannot be excluded. Among the five non-hydrolytic sol-gel condensation steps mentioned above, the following specific reaction paths are of high relevance for the metal oxides: (1) non-hydrolytic hydroxylation reactions; and (2) aprotic condensation reactions.

**Scheme 1:**
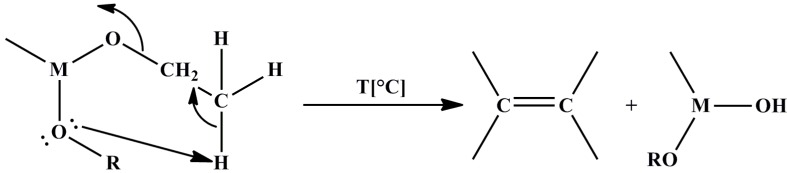
Thermal decomposition of metal alkoxide(carboxylate) precursors.

Thermal decomposition of metal alkoxide(carboxylate) precursors is a non-hydrolytic hydroxylation reaction, where hydroxyl groups are produced via a cyclic elimination mechanism with the liberation of alkene (Scheme 1) [36].

This reaction type is more relevant to MOCVD (metal-organic chemical vapor deposition), but it is probable that it involves the thermal degradation of residual alkoxy groups during the calcinations of non-hydrolytic gels into oxides [36].

**Scheme 2:**
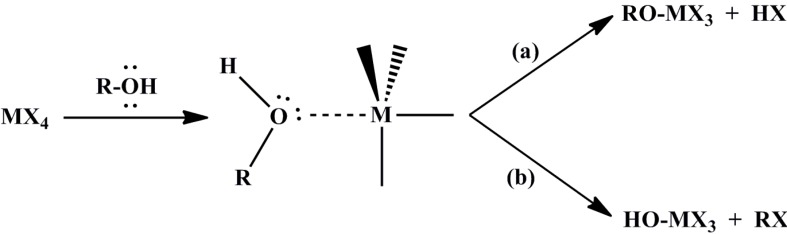
Reaction of different types of alcohols with halides.

Other reactions between primary, secondary and tertiary alcohols and also benzylic alcohols with halides involve, initially, the coordination of a lone pair of electrons of an alcoholic oxygen atom to the metal center, followed by the cleavage of either the hydroxyl or alkoxyl group (Scheme 2). Electron-donor substituent groups in the alkyl radical direct the process to hydroxylation (liberation of RCl) by favoring the nucleophilic attack of chloride on the carbon group, due to its increased cationic character (S_N_^1^ mechanism) [36].

**Scheme 3:**
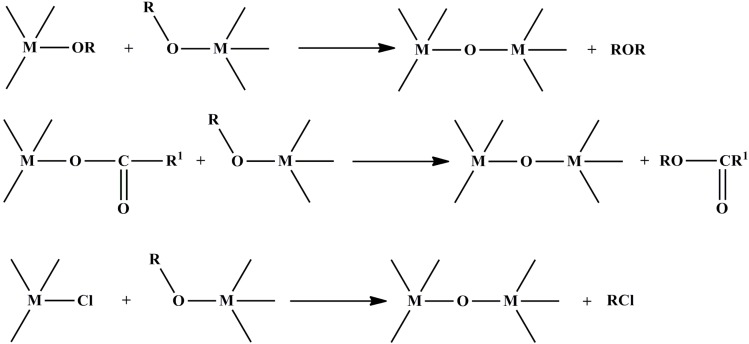
Aprotic condensation reactions.

Aprotic condensation reactions excluding hydroxyl groups are attractive non-hydrolytic methods for the formation of an oxo-bridge normally generated between two different functional groups bonded to two different metal centers by eliminating small organic molecules (Scheme 3) [36].

The advantages of the non-hydrolytic sol-gel method are a direct consequence of the manifold role of the organic components in the reaction system (solvent, organic ligand of the precursor molecule or *in situ* formed organic condensation products). On the one hand, they act as an oxygen-supplier for oxide formation and strongly determine the particle size and shape, as well as the surface properties, due to their coordination properties, and on the other hand, the moderate reactivity of the oxygen-carbon bond generally results in slower reaction rates. Another important point is the fact that the chemistry of the oxygen-carbon bond is well-known in the field of organic chemistry [65].

Summarizing this section, it is important to note that not all wet chemical reactions follow the definition of sol-gel chemistry, and the complex interaction principles on the molecular level have frequently been not fully clarified [36,65].

## 3. Synthesis of n-Type- and p-Type-Like Transition Metal Oxides by Wet Chemical Methods

The sol-gel method for MeO*_x_* is attractive, because it is a low cost atmospheric process, and it can be easily adapted to industrial use. One important argument for using sol-gel synthesis methods is their compatibility for thin film formation from cross-linked liquid chemical precursors by doctor blading, spin-coating, dip-coating or drop-coating. The sol-gel process is a widely accepted method for large-scale architectural coatings. The key to a successful sol-gel method is in the preparation of a stable solution for the deposition.

### 3.1. Zinc Oxide (ZnO)

Zink oxide (ZnO) is an *n*-type, direct, wide band gap (*E*_g_ = 3.37 eV) and non-toxic semiconductor material with attractive electrical [33,66], chemical [44,45,48,52], physical [51,52,67] and magnetic [47] properties. The crystal structures shared by ZnO are wurtzite, zinc blende and rock salt. Under ambient conditions, the thermodynamically stable phase has wurtzite symmetry. The zinc blende ZnO structure can be stabilized only by growth on cubic substrates, and the rock salt, or Rochelle salt (NaCl), structure may be obtained at relatively high pressures [68]. In recent years, ZnO received considerable interest of the scientific community, due to its prospective use as a printable transparent conductive electrode for photovoltaics. Here, the focus will be more on the chemical synthesis of spherical ZnO nanoparticles via the classical sol-gel method and on the preparation of Al^3+^ doped ZnO precursor solutions (sAZO).

#### 3.1.1. Synthesis of Colloidal ZnO Nanoparticles (NPs)

In general, ZnO based on nanocolloids or nanopowders was synthesized by modifying and adapting synthetic routes developed by Bahnemann *et al*. [34], Spanhel *et al*. [35,52], Meulenkamp [37] or Pacholski *et al*. [44]. Depending on the experimental conditions, different types of ZnO nanostructures, such as particles [34,35,37,45] or rods [44], are observed.

The dominant zinc sources are zinc acetate dihydrate, zinc acetate, zinc nitrate and zinc chloride, which are dissolved in alcoholic or other organic solvents as starting solutions. A basic solution containing NaOH, KOH, LiOH H_2_O or LiOH is typically added under vigorous stirring [69], where refluxing of the mixture is optional. It is believed that the formation of colloidal ZnO follows several reaction steps, of which, the last is the transformation of zinc hydroxide to zinc oxide under basic solution conditions. In the presence of acetate ions in the basic solution, the precursors of ZnO are reported to be (1) tetrahedral oxy-acetate Zn_4_O(Ac)_6_ [52], also called “basic zinc acetate”, and its larger self-similar homologue, Zn_10_O_4_(Ac)_12_ [52]; (2) ethoxy acetate (EtOZnAc)*_n_*, forming wire-like nanostructures; and (3) the hydroxyl-acetate Zn_5_(OH)_8_(Ac)_2_∙2H_2_O monomer of lamellar sheet compounds, also known as “hydroxy double salt’’ (Zn-HDS) [52]. It was found that Zn_4_O(Ac)_6_ clusters are formed in ethanol and 1-propanol at temperatures above 50 °C by prolonged refluxing of alcoholic zinc acetate dihydrate (ZAH) salt solutions, and the heating step can be described by the following overall reaction (Equation (2)) [52,70].

4Zn(Ac)_2_∙2H_2_O → Zn_4_O(Ac)_6_ + 7H_2_O + 2HAc (2)

According to Briois *et al*. [70], the formation of Zn-HDS is now recognized for reactions carried out with the addition of water. The following reactions are proposed (Equations 3–5) [52,70]:

Zn_4_O(Ac)_6_ + Zn(Ac)_2_ + 9H_2_O → Zn_5_(OH)_8_(Ac)_2_(H_2_O_2_) + 6HAc (3)

Zn_10_O_4_(Ac)_12_ + 16H_2_O → 2Zn_5_(OH)_8_(Ac)_2_(H_2_O) + 8HAc (4)

Zn_10_O_4_(Ac)_12_ + 8H_2_O + 8OH^−^ → 2Zn_5_(OH)_8_(Ac)_2_(H_2_O_2_) + 8Ac^−^ (5)

In fact, real-time monitoring of chemical reactions taking place in zinc acetate-based precursor solutions reveals a complex chemistry, where the sequences of the formation of ZnO and Zn-HDS are strongly dependent on the oxygen donor source used to initiate the hydrolysis (Briois *et al*. [70]). Using LiOH H_2_O as the basic solution gave the first direct evidence of a reaction between ZnO nanoparticles and unreacted zinc oxy-acetate precursors (Zn_4_O(Ac)_6_) to form the Zn-HDS phase (Equation 6) [70].

Zn_4_O(Ac)_6_ + ZnO + 6H_2_O ⇌ Zn_5_(OH)_8_(Ac)_2_∙2H_2_O + 4HAc (6)

Two effects are responsible for the formation of Zn-HDS: (1) a slow continuous increase of the ZnO concentration and a consequent release of Ac^−^ and water, followed by the hydrolytic attack of precursors and a condensation reaction of zinc hydroxyl species; and (2) the occurrence of ethanol esterification, giving rise also to a slow continuous water release [70].

**Scheme 4:**
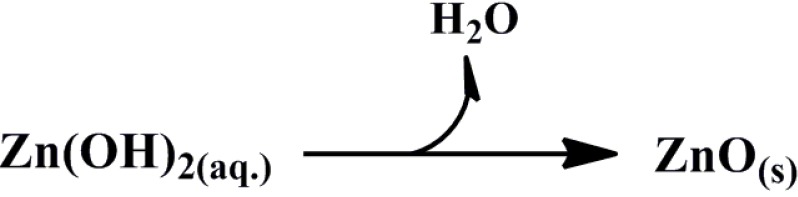
Formation of Zn-O-Zn bridge bonds and precipitation of ZnO via oxolation reaction.

To prevent an increasing H_2_O/ethanol ratio, the formation of secondary phases, like Zn-HDS, and dissolution of “Zn-O-Zn”, we require strong basic conditions, which lead to oxolation reactions, including hydrolysis or condensation forming Zn-O-Zn bridge bonds and precipitation of ZnO (Scheme 4) [70].

In order to prepare zinc oxide nanostructures with different sizes, shapes and morphologies, the reaction temperature can be also varied. After that, zinc oxide precipitate is separated from the reaction mixture by centrifugation and washed with alcohol or deionized water. The performed TEM measurements by Bahnemann *et al*. [34] indicate that the reaction mixture contains almost spherical particles with a mean diameter of 50 Å and a relatively narrow size distribution of (±5 Å). Another approach used by Spanhel *et al*. [35] with refluxing and by Meulenkamp [37] without refluxing led to ZnO nanoparticles in a size range from 2 to 7 nm. Pacholski *et al*. [44] reported the formation of high-quality single crystalline ZnO nanoparticles in a size range of 3 to 5 nm.

All these results confirm that the choice of the anion, the reaction temperature and time, the nature of the alcohol solvents, as well as storage, humidity and analysis conditions are critical for the preparation of transparent, stable ZnO colloids via precipitation sol-gel method [52]. It is also well known that colloidal ZnO nanoparticles (NPs) are not very stable for a long time in solution. Krebs *et al*. [71] proposed the use of ligands, like amine, thiol or a carboxylic acid, to prevent solid zinc oxide from quick precipitation by a polymerization reaction or aggregation of the particles. From the tested ligands mentioned above, alkoxyacetic acids, like methoxyacetic acid (MA), ethoxyacetic acid (MEA) and methoxyethoxyethoxyacetic acid (MEEA), were found to be the best choice for stabilizing ZnO NPs.

Pure ZnO thin films deposited from colloidal ZnO NPs solutions are sensitive to oxidation. Absorption of O_2_ leads to an undesired decrease of the optical and electrical properties of thin films. In order to overcome this limitation and to make ZnO films more stable and suitable for (opto)-electronic applications, many research groups prepared doped ZnO via sol-gel routes [40,43,66]. Group III elements, especially Al^3+^, are good candidates for doping ZnO, because its ionic radius (0.54 Å) is smaller than that of Zn^2+^. Therefore Al^3+^ can occupy the place of Zn^2+^ in the lattice easily [72]. The precipitation approach described above is not suitable for the preparation of Al^3+^-doped ZnO NPs. The reason is that the low-temperature classical sol-gel method used solvents, like MeOH (65 °C), EtOH (78 °C) or i-PrOH (82 °C), which have low thermal energy and, thus, do not provide enough energy to incorporate dopants into the ZnO lattice.

#### 3.1.2. Synthesis of Al^3+^-Doped ZnO Based on Precursor Solutions (sAZO)

One of the most widely reported synthesis routes for Al^3+^-doped ZnO (sAZO) are the methods described by Ohyama *et al*. [40], Alam and Cameron [43]. Synthesis starts in most cases with zinc acetate dihydrate (Zn(Ac)_2_∙2H_2_O) in various organic solvents, like 2-methoxyethanol, isopropyl alcohol or ethanol. To achieve the desired doping, different Al^3+^ precursors, like aluminum nitrate (Al(NO_3_)_3_∙9H_2_O), aluminum acetate (HOAl(C_2_H_3_O_2_)_2_), aluminum chloride (AlCl_3_) or aluminum chloride hydrate (AlCl_3_ 6H_2_O) are dissolved together with zinc acetate dihydrate and an alcohol in the presence of stabilizers, like monoethanolamine (MEA) or diethanolamine (DEA). The resulting solutions are refluxed at 80 °C for two to 3 h to obtain clear, stable and transparent precursor solutions. The same procedure can be used also to prepare pure ZnO precursor solutions without any doping. These precursor solutions can be also directly deposited, but in most cases, a temperature treatment was reported to be essential for the formation of dense MeO*_x_* layers [23,40,43,66].

### 3.2. Titanium Dioxide (TiO_2_)

TiO_2_ is a large band gap semiconductor with three crystalline phases: anatase, rutile and brookite. The most stable phase is rutile, and it is usually obtained after annealing at temperatures above 500 °C. TiO_2_ is transparent in the visible regime; its band gap is 3.02 eV for rutile, 3.2 eV for anatase and 2.96 eV for brookite [73]. The outermost filled orbitals of elemental titanium (Ti) are 4s^2^ and 3d^2^, and that of oxygen (O) are 2s^2^ and 2p^4^. In TiO_2_, the Ti ions are in a distorted octahedral environment and formally have a Ti^4+^ (3d^0^) electronic configuration. The valence band of TiO_2_ is composed primarily of oxygen 2p orbital’s hybridized with Ti 3d states, while the conduction band is purely made up from 3d orbital’s of titanium [73,74,75]. Titanium dioxide (TiO_2_) is widely used as an electron extracting material (EEL) or as a transporting material (ETL) in (opto)-electronic applications.

#### 3.2.1. Synthesis of Colloidal TiO_2_ Nanoparticles (NPs)

N. Pinna and M. Niederberger [56,57] report a number of non-hydrolytic sol-gel synthesis methods, where Ti(O*i*Pr)_4_, TiCl_4_, Ti(O*n*Bu)_4_, Ti(O*t*Bu)_4_ and different combinations of solvents, like toluene, aldehydes, ketones, ethanol, benzyl alcohol, 1,4-butandiol or *n*-butanol, offer a many-sided reaction system for the preparation of crystalline TiO_2_ nanoparticles. Most of these reactions take place in an autoclave, and they are known as solvothermal reactions. One of these solvents, benzyl alcohol, has proven its reliability for the synthesis of crystalline metal oxide nanoparticles in the so-called “benzyl alcohol route’’. This one pot reaction offers controlled metal oxide formation by using various metal complexes (chlorides, alkoxides, acetates, acetylacetonates) in benzyl alcohol, where the latter is acting as a solvent, ligand and reactant [62].

**Scheme 5:**
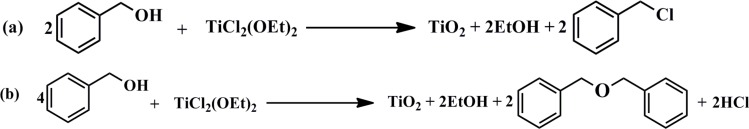
Reaction between TiCl_4_ and benzyl alcohol: (**a**) alkyl halide elimination; and (**b**) ether elimination.

The use of TiCl_4_ as a starting precursor is a common choice for the synthesis of colloidal crystalline TiO_2_ nanoparticles. All reactions can be carried out at temperatures well below the boiling point of the organic solvent in standard laboratory equipment. For the reaction between TiCl_4_ and benzyl alcohol, two possible reaction mechanisms were outlined by Grote *et al*. [61]. The first one presents alkyl halide elimination and the second one, ether elimination (Scheme 5) [61].

Wang *et al*. [55] prepared crystalline TiO_2_ (anatase) nanoparticles using a modified non-hydrolytic sol-gel route reported by Niederberger *et al*. [56], where titanium tetrachloride (TiCl_4_) and benzyl alcohol provide a versatile reaction system for the synthesis of an anatase nanocrystal sol. An advantage of this non-hydrolytic approach is that there is no need for additional peptizing agents, organic ligands or high temperature treatment [57]. The particle size by this method is <10 nm [55].

Jing *et al*. [74] employed a sol-gel method for the synthesis of TiO_2_ nanopowder with Ti(OBu)_4_ as the Ti source. This synthetic route presents a complex approach. First, Ti(OBu)_4_ was mixed with anhydrous ethanol in a dry atmosphere. Then, the mixed Ti(OBu)_4_/ethanol solution was added dropwise into another mixture, which consists of water, anhydrous ethanol and 70% HNO_3_, at room temperature, under vigorous stirring, to carry out a hydrolysis. The yellowish transparent sol was produced after continuously stirring for 3 h. After 6 h at room temperature, the sol was dried at 70 °C for 2 h, and a gel precursor was obtained. Finally, TiO_2_ nanopowders were obtained after the thermal treatment of the gel precursor at a certain temperature for 2 h and a subsequent grinding step [74].

#### 3.2.2. Synthesis of TiO_2_ Based on Precursor Solution (sTiO_2_)

In many cases, commercial titanium(IV)-isopropoxide (Ti(OCH(CH_3_)_2_)_4_) and titanium(IV)-butoxide (Ti(O*n*Bu)_4_) mixed in isopropyl alcohol provide an easy method for the synthesis of the desired TiO_2_ precursor solutions, which can be used for thin film deposition. After film deposition, these precursor solutions typically require a post treatment to form TiO*_x_*. For example, Bolognesi *et al*. [21], using the method described above, received TiO*_x_* thin films only by drying at 90 °C for 5 min.

Kim *et al*. [3] applied a complex synthetic route for the fabrication of TiO*_x_* precursor solution by mixing titanium(IV)-isopropoxide (Ti(OCH(CH_3_)_2_)) with 2-methoxyethanol (CH_3_OCH_2_CH_2_OH) and ethanolamine (H_2_NCH_2_CH_2_OH) in a three-necked flask equipped with a condenser, a thermometer and an argon-gas inlet/outlet. The mixed solution was heated to 80 °C for 2 h, followed by heating to 120 °C for 1 h. These two heating steps (80 and 120 °C) were repeated. The final TiO*_x_* precursor solution was prepared in isopropyl alcohol.

Kuwabara *et al*. [18] prepared TiO*_x_* precursor solutions using the method described by Kim *et al*. [3]. Ethanolamine (EA), diethanolamine (DEA) and acetyl acetone (AA) were utilized as stabilizers. They found that EA-TiO*_x_* and AA-TiO*_x_* precursor solutions took on an orange or yellow color, respectively. The DEA-TiO*_x_* solution was colorless. The coloration for the EA-TiO*_x_* and the AA-TiO*_x_* was explained as the d-d absorption originating from a titanium complex ion formed by the reaction of titanium(IV)-isopropoxide with the stabilizer.

### 3.3. Synthesis of p-Type-Like Transition Metal Oxides by Wet Chemical Methods

Transition *p*-type-like metal oxides MoO_3_, WO_3_ and V_2_O_5_ have various structures and morphologies, which strongly depend on the experimental conditions used. These metals can have different oxidation states, which lead to metal oxides with the same composition and crystal structure, but in general, they exist in various crystal sizes and forms. This is in agreement with the observation that using sol-gel methods to prepare *p*-type-like metal oxides MoO_3_, WO_3_ and V_2_O_5_ allow for the easy variation of the particle morphology. However, synthesis pathways are also much less predictable. In the case of tungsten oxide, the reaction in benzyl alcohol leads to complex lamellar tungsten oxide structures with a lateral size length of 30 to 100 nm [57]. MoO_3_ has been synthesized in several different morphologies, like large-scale α-MoO_3_ nanoplatelets [54], aerogels [38,41], xerogels [38,41] and nanoflowers [59]. Similar observations were reported for V_2_O_5_, where the morphologies were nanobelts, nanorolls [50] or nanorods [46]. The metal oxides mentioned above were chemically and physically characterized, but still more efforts and progress are required to better control the synthesis routes and the conversion of the resulting sol-gel solutions into thin films and electrodes. Therefore, we only highlight those wet chemical methods that were already reported for application in inverted BHJ solar cells.

Liu *et al*. [7] prepared MoO_3_ precursor solutions via the hydration method using ammonium molybdate ((NH_4_)_6_Mo_7_O_24_) dissolved in water. This solution was mixed with a second solution containing hydrochloric acid (HCl) to get a final pH value of 1–1.5. The same procedure could also work in polar organic solvents.

Giroto *et al*. [8] reported bright yellow MoO_3_ solutions, which were stable over several days. MoO_3_ powder was dissolved in H_2_O_2_ and refluxed for 24 h at 80 °C in air and cooled to room temperature (RT) for 24 h to obtain a clear yellow liquid. Furthermore, the viscosity and concentration of the solution was adjusted by the addition of polyethylene glycol and 2-methoxyethanol (volume ratio: 1:0.25:6.25) under refluxing for 30 min at 70 and 60 °C, respectively. When the H_2_O_2_ amount is not enough for the full conversion of MoO_3_ into MoO_2_(OH)(OOH), they observed that the solution changes its color to dark blue [75].

Zilberberg *et al*. [13] reported a fast hydrolysis reaction for sol-gel sMoO*_x_* and sV_2_O_5_ based on precursor solution, where precursor molecules (bis(2,4-pentanedionato)-molybdenum (VI)dioxide) and vanadium(V)-oxytriisopropoxide (OV(OCH(CH_3_)_2_)_3_) are dissolved in isopropanol by stirring at room temperature in ambient atmosphere, respectively. Using vanadium(V)-oxytriisopropoxide (OV(OCH(CH_3_)_2_)_3_) dissolved in anhydrous isopropanol (volume ratio: 1:40), Hancox *et al*. [14] also reported the fabrication of V_2_O*_x_*_(sol)_ layers.

Hammond *et al*. [76] utilized a sMoO*_x_* precursor solution based on metal-organic precursor tricarbonyl trispropionitrile (Mo(CO)_3_(EtCN)_3_) dissolved in acetonitrile. The mixture was stirred at 60 °C for 12–20 h and then filtered.

Choi *et al*. [11], using the same technique, prepared sol-gel sWO_3_ based on precursor solution. The starting molecule, tungsten ethoxide (W(OC_2_H_5_)_6_) was dissolved in ethanol at room temperature to give a clear solution.

At the moment, one of the most frequently used methods for the preparation of high grade pure MoO_3_ or WO_3_ nanoparticles is the so-called “high-temperature hydrolysis process” or flame spray pyrolysis [63]. After that, the particles are dissolved in the desired organic solvent with or without stabilizing molecules to achieve better dispersion properties.

## 4. Requirements for Interface Metal Oxide Materials

In order to better understand which wet chemical pathways are suitable for the preparation of interface metal oxide materials, we summarize the relevant properties of *n*-type- and *p*-type-like metal oxides for application as a charge injection layer in photovoltaic cells. Ginley *et al*. [77] already pointed out that the quality of MeO*_x_* films critically impacts the device performance.

It is well known that MeO*_x_* are wide band gap semiconductors with a relatively high concentration of free electrons in the conduction band. For the present purpose, the MeO*_x_* must carefully balance electrical conductivity and optical transmittance, on the one hand, and stable reproducible chemistry, on the other side. The reduction of the resistivity involves either an increase in the carrier concentration or in the mobility. Increasing the mobility has no negative effect and is most likely the best way to follow [77]. Further, the MeO*_x_* should: (1) favor Ohmic contact formation between electrodes and the active layer; (2) possess suitable energy levels to improve charge selectivity for corresponding electrodes; (3) have a large band gap to confine excitons in the active layer; (4) have sufficient conductivity to reduce resistive losses; (5) have chemical and physical stability to prevent undesirable reactions at the active layer/electrode interface; (6) have the ability to be processed from solution and at low temperatures; (7) be mechanically robust to support multilayer solution processing; (8) have good film forming properties, and, last, but not least, (9) be producible at low cost. At the moment, it is a big challenge to identify metal oxide systems that can simultaneously satisfy all these requirements [78].

The optimization of MeO*_x_* begins with selecting the accurate chemical synthesis methods, which lead to desired products, like nanoparticles or precursor solutions. The classical sol-gel method (precipitation reactions) relies on the formation of metal oxide nanoparticles under strong basic conditions. One disadvantage of this reaction system is the fact that metal oxide particles tend to agglomerate after their dispersion in the desired solvents under the generation of heavy metal hydroxides, like M(OH) or M(OH)_2_.

Metal oxide systems from metal alkoxide precursors (M(OR)*_x_*_ −_*_ y_*) have the advantage that they are soluble in organic solvents, provide homogenous solutions and can easily be converted by a low temperature treatment into the corresponding metal oxide [65].

The nonaqueous or non-hydrolytic sol-gel processes represent an interesting method to overcome some limitations observed by the classical sol-gel method. The success of these approaches is the versatility of the starting precursors in the reaction mixture and the role of the organic solvents (ethers, alcohols, ketones or aldehydes) as supplying oxygen source for the formation of the metal oxide particles [65].

Oh *et al*. [23] successful prepared ZnO NPs using the classical sol-gel method (precipitation reaction), TiO*_x_* NPs applying the nonaqueous sol-gel process and Al^3+^-doped ZnO (sAZO) based on the precursor solution. XRD measurements from those three metal oxides as coated films on glass have confirmed their amorphous structure, but after thermal treatment in air, wurtzite (ZnO NPs, sAZO) and anatase (TiO*_x_* NPs) structures were found. All films of ZnO NPs, sAZO and TiO*_x_* NPs were found to be transparent (over 92%) in the visible range. Also interesting is the wide range of conductivities reported by the same group. They observed a fairly high conductivity for TiO*_x_* NPs films (4.50 × 10^−4^ S/cm) and relatively low conductivity for ZnO NPs films (1.63 × 10^−6^ S/cm). In the case of Al^3+^-doped ZnO (sAZO), annealing had a strong influence on the conductivity, due to the recrystallization. Al^3+^-doped ZnO (sAZO) films annealed at a low temperature (150 °C) had relatively poor conductivity (8.86 × 10^−7^ S/cm) compared to sAZO films (2.35 × 10^−3^ S/cm) annealed at a high temperature (260 °C). Annealing with even higher temperatures resulted in conductivities above 1 S/cm.

Stubhan *et al*. [24] compared the dispersion of ZnO NPs and AZO precursor solution (sAZO). They reported comparable transmittance and device performance for thin ZnO NPs and sAZO layers. Increasing the film thickness (~126 nm) from ZnO NPs dispersions resulted in a significant performance loss, while cells with comparable sAZO film thickness (~119 nm) maintained full performance. Comparable device performance for inverted BHJ solar cells based on ZnO NPs and ZnO precursor solution (sZnO) as the electron transport/extraction layers was reported by Hau *et al*. [17].

An interesting phenomenon related to the use of *n*-type MeO*_x_* layers is the S-shaped current-voltage (*I*-*V*) characteristics, which were reported for MeO*_x_* interface layers from ZnO, TiO_2_ or Al^3+^-doped ZnO. Light soaking under UV radiation is known to release the *S*-shape [79]. The changes in *I*-*V* characteristics and the fill factor (FF (%)) of the inverted solar cells can be correlated with a change in the resistance of the metal oxide thin film. The mechanism behind this is the release of oxygen molecules upon UV irradiation. This results in a decrease in the resistance of the material, due to an increase in the density of mobile charges [79].

Using solution-processed p-type-like transition metal oxides MoO_3_, WO_3_ and V_2_O_5_ as hole extraction/injection layers in inverted BHJ organic solar cells is much more complex. First, they have to be deposited on the top of the photovoltaic active layers, which may cause diverse interactions or chemistry. In addition, post-processing of the sol-gel transition metal oxide layers at high temperatures (300–600 °C), which otherwise is required to receive a specific microstructures or crystalline phases, becomes impossible. Such high temperatures are not compatible with the temperature-sensitive polymers and substrates (foils) for the low-cost fabrication of organic solar cells [10].

Despite all the challenges, low temperature solution processed MoO_3_ and WO_3_ nanoparticle thin films have been reported [9,28]. These particles were prepared by the so-called “high-temperature hydrolysis process” or the flame spray pyrolysis. Furthermore, transition metal oxides based on precursor solutions have been reported, resulting in highly transparent and homogenous solutions [10,11,13,76]. However, the electrical, optical and morphological properties (e.g., electrical conductivity, work function (WF), spectral absorption, *etc*.) of sol-gel processed layers on top of an organic semiconductor are missing [10].

## 5. Development of Inverted BHJ Solar Cell

### 5.1. Device Structure

**Figure 2:**
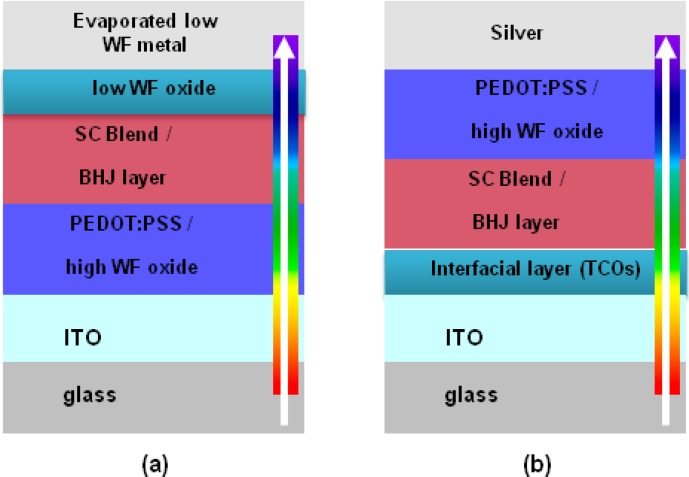
(**a**) Schematic device structure of the conventional polymer solar cell; and (**b**) schematic device structure of the inverted polymer solar cell. SC (Semiconductor); BHJ (inverted bulk heterojunction); PEDOT:PSS (polyethylene dioxythiophene:polystyrene sulfonate); ITO (indium tin oxide); WF (work function).

Today various organic solar cells architectures have been established, such as normal BHJ [3,4,5,6,7,8,9,10,11,12,13,14], inverted BHJ [15,16,17,18,19,20,21,22,23,24,25,26,27,28,29] and tandem solar cells [29]. The normal structure (Figure 2a) includes ITO/polyethylene dioxythiophene:polystyrene sulfonate (PEDOT:PSS)/photoactive layer/low work function(LWF) metal from bottom to top. LWF metal as the cathode is a primary limit to the interface stability of devices, since metals (Li, Ca, and Al) are prone to be oxidized, resulting in the increase of series resistance at the organic/electrode interfaces. In addition, oxygen preferably diffuses into the photoactive layer through pinholes and grain boundaries through the cathode, degrading the active layer and making the device instable in air. On the other hand, the ITO/PEDOT:PSS interface is also instable, due to likely indium diffusion into the photoactive layer and the ITO etching by acidic PEDOT:PSS adsorbing water. The reaction between PSS and water induces a faster degradation of the ITO/PEDOT:PSS interface [2].

To overcome the instability issue in the normal structure device, one feasible approach is to construct an inverted configuration [2,30], where ITO serves as the cathode, as shown in Figure 2b.

It should be pointed out that only modified ITO can serve as the cathode for electron extraction. The functional layers for modifying ITO mainly focus on metal oxides, such as ZnO, Al^3+^-doped ZnO, TiO_2_ and alkali-metal compounds, like Cs_2_CO_3_ [2].

### 5.2. ZnO, Al^3+^-Doped ZnO and TiO_2_ as the Cathode Interlayer in an Inverted Solar Cell

Due to their chemical resistance to oxygen and moisture, optical transparency and facile solution processability, these *n*-type semiconducting oxides effectively replace low work function metals for cathode contacts, resulting in high efficiency devices.

White *et al*. [16] successful applied ZnO based on precursor solution via spin coating as the blocking hole interlayer between the ITO and the active layer (P3HT: PCBM 1:1) using Ag as the hole-collecting back contact. Their inverted architecture with modified ITO shows high external quantum efficiency (EQE), suggesting that the electron mobility of the solution-cast ZnO layer is large enough for fast electron transfer from PCBM to ZnO, with no buildup of electrons at the ZnO/organic interface occurring. They also found that electron transfer from PCBM to ZnO does not result in a significant loss in energy, indicating that the LUMO of PCBM is close to the conduction band energy of ZnO at −4.1 eV.

Hau *et al*. [17] were able to improve the short-circuit current density (*J*_SC_) and open-circuit voltage (*V*_OC_) of inverted polymer solar cells compared to their conventional architecture pendants, by using ZnO based on a colloidal suspension, as well as precursor solutions on both ITO-coated glass substrates and ITO-coated plastic substrates. The devices with ZnO NPs and ZnO precursor solution on ITO-coated glass showed very similar characteristics, with a power conversion efficiency (PCE) of ~3.6%. For the devices produced on ITO-coated plastic, they observed a lower PCE of ~3.3% compared to ITO-coated glass samples, reflecting the lower transparency of the flexible ITO substrates.

Park *et al*. [20] used ZnO as an electron transport buffer layer based on precursor solution deposited via spin coating. After deposition of the ZnO thin films one, two, three, and four times, they observed formation of wrinkles and a little curvature on the metal oxide surface. The device characteristic confirmed that solar cell parameters, like open circuit voltage (*V*_OC_), short circuit current (*J*_SC_) and fill factor (FF), were influenced by the ZnO film thickness and transparency. They claimed the formation of more percolation pathways of the ZnO buffer layer by increasing the thickness.

Oh *et al*. [23], using a ZnO nanoparticles suspension, also found that the performance of the inverted solar cell is strongly dependent on the thickness of the ZnO buffer layer.

Still, there are many problems associated with substituting ZnO-based electron extraction layers. According to Lee *et al*. [27], low-temperature synthesized ZnO thin films have low quality and low optical properties, due to the unexpected defects. In some cases, devices based on colloidal ZnO NPs require UV light treatment to improve their conductivity. This effect was tested by Lee *et al*. [27] in the device structure of ITO/ZnO/P3HT:PCBM/MoO_3_/Au, and the experimental results showed that the transmittance of the ZnO thin layer increased after UV light illumination, followed by an increase in the open circuit current (*J*_SC_), while the values for the open circuit voltage (*V*_OC_) remained almost constant [27]. However, according to Chen *et al*. [30], this photo-doping effect only lasts for a short time. In order to enhance the stability of colloidal ZnO NPs solutions, Krebs *et al*. [71] proposed the use of ligands to prevent aggregation and to stabilize the zinc oxide nanoparticles.

One of the most used chemical approaches to enhance device performance is to effectively dope ZnO with trivalent elements from the group III (Al, Ga, In). The ionic radius of Al^3+^ is 0.54 Å and is smaller than that of Zn^2+^ (0.74 Å), allowing Al^3+^ to substitute Zn^2+^ in the lattice. The resistivity of the AZO film decreased with an increase of the Al content up to a few atomic percentages (at%). At a high Al^3+^ concentration, Al^3+^ exists in the form of Al_2_O_3_, resulting in the deterioration of electrical properties [72].

Sol-gel based methods have been established for the fabrication of inverted solar cells based on Al^3+^-doped ZnO. Oh *et al*. [23] fabricated efficient solar cells organic solar cells with an inverted device geometry, where sAZO precursor solution with different Al^3+^ content (0.5, 1 and 2 at %) was coated onto ITO-glass substrates as a bottom electrode interlayer. They concluded that the variation of the doping degree between 0.5% and 2% did not make a major impact on the device performance. The resulting device performance was influenced by the annealing temperature, and the obtained PCEs were between 2.18% and 2.59%.

Stubhan *et al*. [24] compared a dispersion of i-ZnO nanoparticles (ZnO NPs) and a sAZO precursor solution, as electron injection layers. Increasing the film thicknesses of both materials showed that the performance of the sAZO inverted solar cell did not change with increasing film thickness compared to i-ZnO nanoparticle thin films. The conductivity of sAZO thin films was reported to be one to two orders of magnitude higher than that for i-ZnO films.

TiO_2_ as the electron collection electrode in inverted BHJ solar cells was reported by Waldauf *et al*. [15]. They prepared inverted devices (ITO/TiO*_x_*/RR-P3HT:PCBM/PEDOT:PSS/Au) via coating techniques and demonstrated improved fill factors (FF, 62%) for the inverted architecture compared to the conventional architecture with an FF of 59%. Kuwabara *et al*. [18] reported efficient inverted BHJ solar cells by comparing different types of TiO*_x_* precursor solutions. They observed an improved short-circuit photocurrent (*J*_SC_), open-circuit voltage (*V*_OC_), fill factor (FF%) and the power conversion efficiency (η). Oh *et al*. [23] also reported an efficient inverted BHJ solar cell based on optimized TiO*_x_* NP solution as the electron extraction layer.

Thin transparent electrodes based on TiO_2_ solutions were also propagated as an optical spacer for improving light absorption in the active film. For thin active layers, Kim *et al*. [3] and Roy *et al*. [5] reported higher short current densities, due to an improved light management with a TiO_2_ electron extracting layer.

The importance of the interface materials is demonstrated even more powerfully for the third generation of polymeric semiconductors, like poly[N-9"-hepta-decanyl-2,7-carbazole-alt-5,5-(4',7'-di-2-thienyl-2',1',3'-benzothiadiazole)] (PCDTBT), dithienosilole-thienopyrrole-4,6-dione (PDTS-TPD) or dithienogermole-thienopyrrole-4,6-dione, which are more air-stable photoactive materials compared to the poly(3-hexylthiophene) (P3HT) high-efficiency solar cells that are fabricated. For example, Sun *et al*. [25] demonstrated an efficient inverted BHJ solar cell based on sol-gel-derived ZnO as a bottom cathode and reported efficiencies of up to 6.08%.

The performance parameters (*V*_OC_ (V); *J*_SC_ (mA/cm^2^); FF (%); PCE (%)) of ZnO, Al^3+^-doped ZnO and TiO_2_ used as the cathode interlayers in inverted organic BHJ solar cells are tabulated in the following Table 1.

**Table 1:** Device parameters of i-BHJ solar cells with different electron transport/extraction interlayers. EEL, electron extraction layer; ETL, electron transport layer; *V*_OC_, open-circuit voltage; *J*_SC_, short-circuit current density; FF, fill factor; NP, nanoparticle.

Reference (N°)	EEL/ETL	*V*_OC_ (V)	*J*_SC_ (mA/cm^2^)	FF%	PCE%
[17] (P3HT:PCBM)	ZnO NP	0.62	11.17	54.3	3.78
[23] (P3HT:PCBM)	ZnO NP	0.56	8.33	56.5	2.62
[24] (P3HT:PCBM)	ZnO NP	0.56	8.36	53.9	2.56
[16] (P3HT:PCBM)	s(ZnO)	0.55	9.23	51.8	2.65
[20] (P3HT:PCBM)	s(ZnO)	0.55	8.86	45.5	2.21
[23] (P3HT:PCBM)	s(AZO)	0.57	9.57	47.7	2.59
[24] (P3HT:PCBM)	s(AZO)	0.57	8.36	50.8	2.42
[23] (P3HT:PCBM)	TiO_2_∙NP	0.57	8.38	54.4	2.58
[18] (P3HT:PCBM)	s(EA-TiO*_x_*)	0.56	6.70	55.0	2.06
[18] (P3HT:PCBM)	s(DEA-TiO*_x_*)	0.55	5.31	36.0	1.06
[18] (P3HT:PCBM)	s(AA-TiO*_x_*)	0.55	7.00	60.0	2.31
[21] (P3HT:PCBM)	sTiO_2_	0.55	9.06	51.9	2.61

### 5.3. MoO_3_, WO_3_ and V_2_O_5_ as the Anode Interlayer in an Inverted Solar Cell

The binary MeO*_x_*, such as MoO_3_, WO_3_ and V_2_O_5_, are *n*-type oxides. Due to their low lying conduction band, these MeO*_x_* were reported to *p*-dope most organic semiconductors, which makes them suitable as hole extraction layers. Here, we will focus on solution processed MoO_3_, WO_3_ and V_2_O_5_ thin films as hole extraction/injection layers in inverted BHJ solar cells. At the moment, the most widely used hole injection/extraction layers (HIL/HEL) in organic photovoltaic are based on polyethylene dioxythiophene:polystyrene sulfonate (PEDOT:PSS). According to Meyer *et al*. [31], the aqueous PEDOT:PSS solution and its acidic nature can cause the degradation of subsequently deposited organic films or electrode materials. In addition, PEDOT:PSS has a relatively low WF of 5.0 eV, which can limit charge extraction from deep HOMO polymers. In comparison, experimental PES/IPES measurement, outlined also by Meyer *et al*. [31,80], showed that V_2_O_5_ has the largest WF (7.0 eV), closely followed by MoO_3_ and WO_3_ with WF values of 6.9 eV and 6.7 eV, respectively. Another interesting point is the fact that MeO*_x_*, like MoO_3_, WO_3_ and V_2_O_5_, do not act as the electron blocking layer, because their conduction band (CB) edge is too low [31,80,81].

However, access to solution-based *p*-type-like metal oxides open the way to replacing PEDOT:PSS as the hole-injection or hole-extraction layer and to improving the performance of the organic solar cells.

Zilberberg *et al*. [26] reported an inverted BHJ solar cells (TCO/TiO*_x_*/P3HT:PCBM/sV_2_O_5_/Ag) based on sV_2_O_5_ spin-coated precursor films as hole-extraction layers. They found that the thickness of sV_2_O_5_ thin film critically influences solar cell performance. Comparable *V*_OC_ values for an inverted solar cell with a solution-processed sV_2_O_5_ layer and with an evaporated eV_2_O_5_ layer were observed. Using the same preparation method for sVO*_x_* precursor solution (VTIPO (vanadium (V) triisopropoxy oxide)), Chen *et al*. [22] fabricated an inverted BHJ solar cell with the layered configuration of glass/ITO/ZnO/polymer (P3HT or a-PTPTBT):PCBM/sV_2_O_5_ or VO*_x_*/Ag, where V_2_O_5_ powder was homogenously dispersed and suspended in isopropyl alcohol (IPA). They suggested that VO*_x_* layers based on precursor solutions provided improved device characteristic for both organic polymers used (P3HT or poly(thiophene-phenylene-thiophene)-(2,1,3-benzothiadiazole) (a-PTPTBT)) compared to solar cells with sV_2_O_5_ and PEDOT:PSS.

Sun *et al*. [25] combined both cathode and anode interface layer ZnO and evaporated MoO_3_ to prepare efficient inverted BHJ solar cell based on PCDTBT:PC_70_BM. Their investigations focused on the annealing temperatures of ZnO and concluded that 150 °C or 200 °C annealed layers show higher performance (PCE ~6%) than layers annealed at 130 °C. Solution-processed sMoO_3_ as a PEDOT:PSS replacement was reported by Stubhan *et al*. [9] for a normal architecture device (Glass/ITO/sMoO_3_/P3HT:PCBM/Al). They observed that the solar cell performance and, here, especially, the short circuit current (*J*_SC_) were critically influenced by three effects, depending on the sMoO_3_ layer thickness (optical losses, serial resistance (R_S_) and surface roughness). Tungsten oxide (WO_3_) formed by sol-gel chemistry was also successful applied as PEDOT:PSS replacement in a conventional device by Choi *et al*. [11].

Ning *et al*. [28,29] and Stubhan *et al*. [12] finally demonstrated that sWO_3_ based on nanoparticle solutions does show full device performance in inverted organic photovoltaic (OPV) devices (ITO/Al-ZnO/P3HT or Si-PCPDTBT/sWO_3_ NP/Ag). The same conclusion as previously drawn by Stubhan *et al*. [9] for sMoO_3_, namely, the critical importance of film quality was also reported in this work.

The performance parameters: *V*_OC_ (V); *J*_SC_ (mA/cm^2^); FF (%); PCE (%) of MoO_3_, WO_3_ and V_2_O_5_ used as anode interlayers in inverted or conventional organic BHJ solar cells are summarized in the following Table 2.

**Table 2:** Device parameters of i-BHJ solar cells with different hole transport/extraction interlayers. HEL, hole extraction layer; HTL, hole transport layer.

Reference (N°)	HEL/HTL	*V*_OC_ (V)	*J*_SC_ (mA/cm^2^)	FF%	PCE%
[26] (P3HT:PCBM)	V_2_O_5_ NP	0.56	10.4	66.0	3.80
[26] (P3HT:PCBM)	sV_2_O_5_	0.52	9.50	60.0	3.00
[22] (P3HT:PCBM)	sVO*_x_*	0.57	10.1	67.0	3.90
[20] (P3HT:PCBM)	eMoO_3_	0.55	8.86	45.5	2.21
[25] (PCDTBT:PC_70_BM)	eMoO_3_	0.88	10.4	68.8	6.08
[9] *con.* (P3HT:PCBM)	sMoO_3_	0.57	7.96	66.7	2.92
[11] *con.* (P3HT:PCBM)	sWO_3_	0.62	8.63	63.0	3.37
[28] (P3HT/PCDTBT:PCBM)	sWO_3_	0.53	8.56	52.6	2.68
[12] (P3HT/PCDTBT:PCBM)	sWO_3_	0.54	8.50	51.3	2.40

## 6. Device Stability

The development of stable and efficient inverted BHJ solar cells requires intensive device stability testing. Most studies, however, focus on the fundamental degradation mechanisms of the organic semiconductors, rather than the interfaces [10,17,82]. It is well known that oxygen and moisture lead to the degradation of the polymers and polymer/oxide composites. The inverted solar cell architecture clearly bears the advantage of separating the acidic poly (3,4-ethylenedioxythiophene):poly(styrenesulfonate) (PEDOT:PSS) from the indium tin oxide (ITO) electrode and of replacing non-noble metal cathodes. The additional positive impact of MeO*_x_* on the lifetime of organic solar cells is currently under discussion.

Kuwabara *et al *. [18] performed durability tests on ITO/TiO*_x_*/PCBM:P3HT/PEDOT:PSS/Au inverted-type BHJ organic solar cells in an ambient atmosphere. They observed that the solar cells with amorphous TiO*_x_* had high durability under continuous irradiation for 120 h in air (efficiency decrease by approximately 70%), while sealed devices maintained efficiency under continuous light irradiation for 120 h.

Hau *et al*. [17] explored the stability of ITO/ZnO NPs/P3HT:PCBM/PEDOT:PSS/Ag solar cells, where the samples were stored in air under ambient conditions for 40 days. For the conventional devices based on LiF/Al electrodes, they observed drastic degradation after one day of storage, and after four days, the cells were completely degraded. In contrast, inverted-type cells showed relatively constant FF and *V*_OC_ values over the period of 40 days, with only slight *J*_SC_ reduction. The improved lifetime was correlated to the use of an air-stable Ag top electrode.

White *et al*. [16] tested device durability by measuring the performance of an inverted solar cell (ITO/sZNO/P3HT:PCBM/Ag) after different periodic exposures to air. Four days after fabrication, the device efficiency dropped to η = 2.58% from η = 2.97%, and after seven days, the efficiency was η = 2.32%. They realized that the whole device performance improved upon exposure of the ZnO and Ag surface to air, but the P3HT:PCBM layer was responsible for the observed degradation under ambient atmosphere.

Hauch *et al*. [82] applied the accelerated lifetime (ALT) test with conditions close to international standards for inorganic solar cells. Further, using this degradation test, they investigated the degradation of flexible encapsulated P3HT:PCBM cells by three different climate conditions: 65 °C (high T dark storage), 65 °C/1 sun (sun soak) and 65 °C/85% relative humidity (rh) (damp heat). They determined that different environmental stress conditions led to different types of degradation behavior, which also differently influenced the solar cell parameters, like *V*_OC_, *J*_SC_, FF and efficiency (η) [82]. With this experimental data, they were able to show that P3HT:PCBM cells are considerably less sensitive to water and oxygen, as expected.

Sun *et al*. [25] exposed the following inverted cell architecture, ITO/sZnO/PCDTBT:PC_70_BM/MoO*_x_*/Ag, continuously to air at room temperature without encapsulation for 30 days. The cells with MoO*_x_* as the hole transport layer showed PCEs of 70% of its original value after 30 days.

## 7. Summary and Outlook

In the past couple of years, numerous wet chemical synthesis methods for nanoscale metal oxides have been described. Such synthesis routes provide access to nanoparticles with different chemical compositions, monodisperse crystallite sizes, demanding crystal forms and complex configurations. There is no doubt that the physical and chemical properties of the synthesized metal oxide nanoparticles, compared to synthesis pathways, require further detailed work and investigation. From this point of view, in-depth studies of synthesis and product characteristics are an important step on the way to implementing faster, new technological applications.

The non-aqueous sol-gel methods, compared to classical sol-gel methods, offer the possibility for stable synthesis methods for metal oxide nanoparticles. Due to their simplicity and robustness, the reaction conditions exhibit only slight deviations, which typically do not impact the morphological properties of the nanoparticles.

Despite all the efforts and the enormous progress in the field of nanoparticle research, the prime goal is and remains the development of new synthesis strategies for metal oxide particles, allowing one to precisely predict the composition, structure, size, form and opto-electronic properties.

Metal oxide semiconductor nanocrystals based on sol-gel synthesis routes, like ZnO, Al^3+^-doped ZnO, TiO_2_, s-WO_3_, s-MoO_3_ and s-V_2_O_5_, have attracted intensive interest during the last 10 years, because their outstanding optical and electrical properties qualify them for multiple opto-electronic applications, like flat screens, touch screens, sensors, photodetectors or thin film solar cells. To realize such large-scale production of these layers, we require cost-effective synthesis methods, which provide high quality layers.

Research in the field of the solution-processed organic polymer solar cells has already demonstrated that using i-ZnO, Al^3+^-doped ZnO or TiO_2_ as electron transport/extraction interfaces and WO_3_, MoO_3_ or V_2_O_5_ as hole transport/extraction interlayers are viable strategies. Efficient solar cells with superior environmental stability were reported with MeO*_x_* interface layers. Table 1 and Table 2 in Section 5 summarize, once again, the performance parameters (*V*_OC _(V); *J*_SC_ (mA/cm^2^); FF (%); PCE (%)) for various inverted organic BHJ solar cells.

Interestingly, many different synthesis routes for the various MeO*_x_* yielded more or less identical device functionality and performance. Despite the efforts of the last few years, there is still insufficient understanding of how to correlate the detailed chemical and physical properties of thin sol-gel MeO*_x_* to solar cell performance. Light soaking, current mismatching or the observed degradation trends underline that the precise function of MeO*_x_* layers and their impact on the degradation mechanism is not understood. More fundamental investigation on the pristine layers is required to further enhance the intrinsic stability of MeO*_x_* thin films and to pave the way for the fabrication of more efficient and stable organic BHJ solar cells.
